# Global assessment of arteriolar, venular and capillary changes in normal tension glaucoma

**DOI:** 10.1038/s41598-020-75784-1

**Published:** 2020-11-05

**Authors:** Timothy P. H. Lin, Yu Meng Wang, Kevin Ho, Cherie Y. K. Wong, Poemen P. Chan, Mandy O. M. Wong, Noel C. Y. Chan, Fangyao Tang, Alexander Lam, Dexter Y. L. Leung, Tien Yin Wong, Ching-Yu Cheng, Carol Y. Cheung, Clement C. Tham

**Affiliations:** 1 Department of Ophthalmology and Visual Sciences, The Chinese University of Hong Kong, Hong Kong, China; 2 Hong Kong Eye Hospital, Hong Kong, China; 3 Department of Ophthalmology, Prince of Wales Hospital, Hong Kong, China; 4 Department of Ophthalmology, Hong Kong Sanatorium and Hospital, Hong Kong, China; 5 Singapore Eye Research Institute, Singapore National Eye Center, Singapore, Singapore

**Keywords:** Medical research, Translational research, Optic nerve diseases, Glaucoma

## Abstract

Microcirculatory insufficiency has been hypothesized in glaucoma pathogenesis. There is a scarcity of data to comprehensively examine the changes in retinal microvasculature and its role in normal tension glaucoma (NTG). We conducted a cross-sectional case–control study and included 168 eyes from 100 NTG patients and 68 healthy subjects. Quantitative retinal arteriolar and venular metrics were measured from retinal photographs using a computer-assisted program. Radial peripapillary capillary network was imaged with OCT-A and quantitative capillary metrics (circumpapillary vessel density (cpVD) and circumpapillary fractal dimension (cpFD)) were measured with a customized MATLAB program. We found that NTG was associated with decreased arteriolar and venular tortuosity, arteriolar branching angle, cpVD and cpFD. Decreased venular caliber, arteriolar and venular branching angles, cpVD and cpFD were associated with thinner average RNFL thickness. Decreased arteriolar and venular branching angles, cpVD and cpFD were also associated with worse standard automated perimetry measurements (mean deviation and visual field index). Compared with retinal arteriolar and venular metrics, regression models based on OCT-A capillary metrics consistently showed stronger associations with NTG and structural and functional measurements in NTG. We concluded that NTG eyes showed generalized microvascular attenuations, in which OCT-A capillary metrics attenuations were more prominent and strongly associated with NTG.

## Introduction

Glaucoma, a major cause of irreversible blindness in the world, is a progressive optic neuropathy characterized by gradual degeneration of retinal ganglion cells (RGCs)^1,2^. While elevation of intraocular pressure (IOP) is a major risk factor of glaucoma^3^, it is increasingly recognized that many patients with primary open angle glaucoma (POAG) have IOP within the normal range^4^. Such phenomenon of normal tension glaucoma (NTG) constituting the majority of POAG cases (77–92%) is particularly common among Asian populations^5,6^. The etiology of NTG remains largely unknown and current literature suggests a role of vascular dysfunction in the pathogenic process^5,7,8^. The prevailing hypothesis is that in the absence of elevated IOP, compared with high-tension glaucoma, microcirculatory insufficiency may play a more pivotal role in NTG pathogenesis^9–12^.

The non-invasive assessment of microvascular changes in the retina has evolved. Previously, quantitative retinal microvasculature analysis was confined to arterioles and venules (100–300 µm in diameter) based on fundus photography. Studies using this technique have reported that eyes with POAG have decreased retinal vessel caliber, tortuosity, branching angle and fractal dimension^13–15^, and eyes with thinner retinal nerve fiber layer (RNFL) have decreased retinal vessel caliber and fractal dimension^16–18^. Increasingly, capillary network (5–6 µm in diameter) can be analysed using optical coherence tomography angiography (OCT-A)^19,20^. OCT-A studies have similarly reported decreased circumpapillary vessel density (cpVD), quantified from radial peripapillary capillary network, in eyes with POAG^21–23^. Nevertheless, previous studies have focused on and utilized only either of these modalities, and a comprehensive assessment of global microvascular changes (arteriolar, venular, capillary) in glaucoma is lacking. Furthermore, studies specifically on NTG vasculature remain scarce. There is one study (n = 28) reporting no differences in retinal vessel caliber measured from retinal photographs in NTG eyes as compared with normal subjects^24^, which conflicts results of previous studies from comparison between POAG and normal eyes showing reduced vessel caliber in POAG^13,14^, and only a few OCT-A studies with relatively small sample sizes (n ≤ 31) reporting reduced cpVD in NTG, compared with normal subjects^25–28^.

The objective of this study was two-fold. First, we aimed to offer a comprehensive global assessment of microvascular changes (arteriolar, venular and capillary) in NTG, and the associations between such vascular changes with glaucoma-related structural and functional changes in NTG. Second, we aimed to compare the relative associations of arteriolar and venular changes with capillary changes in NTG eyes with the underlying structural and functional changes. The capillary network is the site of tissue perfusion for oxygen delivery and waste removal. Any vascular insufficiency and hypoxia–ischemia injury in NTG should therefore have direct impacts to the capillary network which is adjacent to the site of primary insults and hence be best reflected by changes and dysfunction of the capillary network. We therefore hypothesized that NTG eyes have global microvascular attenuations and that capillary metrics are more prominent and important microvascular markers.

## Results

At baseline, 17 study subjects (15 NTG patients and 2 normal controls) were excluded because 6 of them had ungradable retinal photographs and 11 of them had ungradable OCT-A images. The remaining 168 subjects were included in the final analysis. The characteristics of included study subjects were shown in Table 1. NTG patients were predominantly male and more likely to have history of obstructive sleep apnea (OSA) (8% vs. 0%) compared with normal control subjects (both *P* < 0.05). NTG patients were also more myopic (− 3.19 D vs. − 0.69 D), had longer axial length (25.53 mm vs. 24.08 mm), worse SAP MD (− 6.73 dB vs. 0.18 dB), worse SAP PSD (7.41 dB vs. 1.62 dB), worse SAP VFI (82.42% vs. 99.29%) and thinner average RNFL thickness (72.41 µm vs. 95.04 µm) compared with normal control subjects (all *P* < 0.001). There were no significant differences in age, history of hypertension, cerebrovascular accident and ischemic heart disease, SBP, DBP, IOP and CCT between NTG patients and normal control subjects (all *P* > 0.05). The comparison of retinal photographic arteriolar and venular metrics and OCT-A capillary metrics between NTG and normal eyes were shown in Supplementary Table S1 online.

**Table 1:** Demographics and ocular characteristics of study population.

	Normal (n = 68)	NTG (n = 100)	*P* value*
Eyes (n)	68	100	
Sex (M/F)	20/48	53/47	0.002
Age at recruitment (years)	57.71 (11.09)	58.86 (12.0)	0.097
Hypertension, n (%)	21 (30.88%)	31 (31%)	0.987
Obstructive sleep apnea, n (%)	0 (0%)	8 (8%)	0.017
Cerebrovascular accident, n (%)	3 (4.41%)	1 (1%)	0.26
Ischaemic heart disease, n (%)	6 (8.82%)	6 (6%)	0.485
SBP (mmHg)	139.57 (21.58)	134.74 (20.26)	0.141
DBP (mmHg)	81.71 (12.07)	80.18 (9.91)	0.372
IOP (mmHg)	14.07 (2.93)	14.88 (2.95)	0.084
CCT (microns)	543.87 (34.25)	535.4 (33.03)	0.11
Spherical errors (D)	− 0.69 (2.52)	− 3.19 (3.87)	< 0.001
Axial length (mm)	24.08 (1.23)	25.53 (1.76)	< 0.001
SAP MD (dB)	0.18 (0.90)	− 6.73 (5.49)	< 0.001
SAP PSD (dB)	1.62 (0.34)	7.41 (4.11)	< 0.001
SAP VFI (%)	99.29 (0.83)	82.42 (16.64)	< 0.001
Average RNFL thickness, Cirrus (μm)	95.04 (9.58)	72.41 (10.24)	< 0.001

*CCT* central corneal thickness; *DBP* diastolic blood pressure; *IOP* intraocular pressure; *MD* mean deviation; *NTG* normal tension glaucoma; *PSD* pattern standard deviation; *VFI* visual field index; *RNFL* retinal nerve fiber layer; *SAP* standard automated perimetry; *SBP* systolic blood pressure.

*Statistical significance tested by independent samples *t* test (for continuous variables) or Chi-square test (for categorical variables).

Table 2 shows the odds of NTG in relationship to decreased measurements in retinal photographic arteriolar and venular and OCT-A capillary metrics. After adjusting for age, sex, AL, SBP, fellow caliber (for arteriolar and venular calibers) and OCT-A quality score (for OCT-A capillary metrics), straighter arterioles and venules were both associated with NTG (OR, 1.71; CI, 1.15–2.52 and OR, 1.73; CI, 1.15–2.59 for arteriolar and venular tortuosity, respectively). Narrower arteriolar branching angle was also associated NTG (OR, 1.52; CI, 1.01–2.30). Lower cpVD and cpFD were both associated with NTG (OR, 2.77; CI, 1.64–4.69 and OR, 8.80; CI, 3.93–19.70 for cpVD and cpFD, respectively).

**Table 2:** Relationship of retinal photographic and OCT-A microvascular metrics with NTG.

Retinal microvascular metrics		Univariable		Multivariable	
		NTG, OR (95% CI)	*P* value	NTG, OR (95% CI)	*P* value
**Photographic vascular metrics**					
CRAE	Per SD decrease	1.63 (1.17–2.28)	0.004	0.86 (0.49–1.53)	0.612
CRVE	Per SD decrease	1.70 (1.22–2.38)	0.002	1.47 (0.85–2.56)	0.168
Arteriolar D_f_	Per SD decrease	1.21 (0.89–1.67)	0.228	0.95 (0.62–1.44)	0.799
Venular D_f_	Per SD decrease	1.09 (0.79–1.47)	0.628	1.04 (0.70–1.53)	0.851
Arteriolar tortuosity	Per SD decrease	1.41 (1.03–1.94)	0.032	1.71 (1.15–2.52)	0.007
Venular tortuosity	Per SD decrease	1.25 (0.92–1.71)	0.154	1.73 (1.15–2.59)	0.008
Arteriolar branching angle	Per SD decrease	1.73 (1.22–2.46)	0.002	1.52 (1.01–2.30)	0.046
Venular branching angle	Per SD decrease	1.37 (1.00–1.89)	0.054	1.39 (0.96–2.01)	0.083
**OCT-A capillary metrics**					
cpVD	Per SD decrease	2.27 (1.53–3.37)	< 0.001	2.77 (1.64–4.69)	< 0.001
cpFD	Per SD decrease	5.14 (2.88–9.17)	< 0.001	8.80 (3.93–19.70)	< 0.001

*CI* confidence interval; *CRAE* central retinal arteriolar equivalent; *CRVE* central retinal venular equivalent; *cpVD* circumpapillary vessel density; *cpFD* circumpapillary fractal dimension; *D*_*f*_ fractal dimension; *NT*G normal tension glaucoma; *OR* odds ratio; *SD* standard deviation.

Multivariable model adjusted for age, sex, axial length, systolic blood pressure, fellow caliber (for CRAE and CRVE) and OCT-A image quality score (for OCT-A capillary metrics).

The relationships between the microvascular imaging metrics and average RNFL thickness were summarized in Table 3. In the multivariable model, each SD decrease in venular caliber, arteriolar branching angle, venular branching angle, cpVD and cpFD were associated with 3.22 µm (*P* = 0.04), 2.78 µm (*P* = 0.011), 2.69 µm (*P* = 0.01), 4.95 µm (*P* < 0.001) and 5.52 µm (*P* < 0.001) decrease in average RNFL thickness, respectively.

**Table 3:** Relationship of retinal photographic and OCT-A microvascular metrics with average RNFL thickness.

Retinal microvascular metrics		Univariable		Multivariable	
		Average RNFL thickness, m (95% CI)	*P* value	Average RNFL thickness, μm (95% CI)	*P* value
**Photographic vascular metrics**					
CRAE	Per SD decrease	− 4.81 (− 6.98 to − 2.65)	< 0.001	− 0.44 (− 3.68 to 2.80)	0.788
CRVE	Per SD decrease	− 5.33 (− 7.47 to − 3.19)	< 0.001	− 3.22 (− 6.29 to − 0.14)	0.04
Arteriolar D_f_	Per SD decrease	− 1.90 (− 4.17 to 0.37)	0.1	0.31 (− 2.11 to 2.73)	0.8
Venular D_f_	Per SD decrease	− 1.47 (− 3.74 to 0.81)	0.206	− 0.82 (− 3.06 to 1.41)	0.468
Arteriolar tortuosity	Per SD decrease	− 0.90 (− 3.19 to 1.38)	0.436	− 1.02 (− 3.10 to 1.06)	0.333
Venular tortuosity	Per SD decrease	0.83 (− 1.46 to 3.11)	0.476	− 0.01 (− 2.11 to 2.10)	0.995
Arteriolar branching angle	Per SD decrease	− 4.48 (− 6.66 to − 2.30)	< 0.001	− 2.78 (− 4.90 to − 0.65)	0.011
Venular branching angle	Per SD decrease	− 2.89 (− 5.14 to − 0.65)	0.012	− 2.69 (− 4.72 to − 0.66)	0.01
**OCT-A capillary metrics**					
cpVD	Per SD decrease	− 5.43 (− 7.56 to − 3.30)	< 0.001	− 4.95 (− 7.05 to − 2.85)	< 0.001
cpFD	Per SD decrease	− 6.08 (− 8.17 to − 3.99)	< 0.001	− 5.52 (− 7.51 to − 3.54)	< 0.001

*CI* confidence interval; *CRAE* central retinal arteriolar equivalent; *CRVE* central retinal venular equivalent; *cpVD* circumpapillary vessel density; *cpFD* circumpapillary fractal dimension; *D*_*f*_ fractal dimension; *RNFL* retinal nerve fiber layer; *SD* standard deviation.

Multivariable model adjusted for age, sex, axial length, systolic blood pressure, fellow caliber (for CRAE and CRVE), OCT signal strength and OCT-A image quality score (for OCT-A capillary metrics).

The relationships between the microvascular imaging metrics and SAP measurements (MD and VFI) were summarized in Table 4. In the multivariable model, each SD decrease in arteriolar branching angle was associated with 1.10 dB decrease in MD (*P* = 0.007) and 2.85% decrease in VFI (*P* = 0.014), respectively. Each SD decrease in venular branching angle was associated with 0.96 dB decrease in MD (*P* = 0.015) and 2.67% decrease in VFI (*P* = 0.017), respectively. Each SD decrease in cpVD was associated with 2.34 dB decrease in MD (*P* < 0.001) and 6.46% decrease in VFI (*P* < 0.001), respectively. Each SD decrease in cpFD was associated with 2.48 dB decrease in MD (*P* < 0.001) and 7.36% decrease in VFI (*P* < 0.001), respectively.

**Table 4:** Relationship of retinal photographic and OCT-A microvascular metrics with standard automated perimetry functional measurements.

Retinal microvascular metrics		Univariable				Multivariable			
		SAP MD, dB (95% CI)	*P* value	SAP VFI, % (95% CI)	*P* value	SAP MD, dB (95% CI)	*P* value	SAP VFI, % (95% CI)	*P* value
**Photographic vascular metrics**									
CRAE	Per SD decrease	− 1.07 (− 1.89 to − 0.25)	0.011	− 2.91 (− 5.21 to − 0.61)	0.013	− 0.30 (− 1.53 to 0.92)	0.627	− 1.93 (− 5.41 to 1.55)	0.275
CRVE	Per SD decrease	− 1.00 (− 1.82 to − 0.17)	0.018	− 2.31 (− 4.63 to 0.004)	0.05	− 0.23 (− 1.41 to 0.96)	0.704	0.14 (− 3.22 to 3.50)	0.933
Arteriolar D_f_	Per SD decrease	− 1.06 (− 1.88 to 0.24)	0.011	− 2.55 (− 4.86 to − 0.24)	0.031	− 0.85 (− 1.76 to 0.50)	0.063	− 2.40 (− 4.96 to 0.17)	0.067
Venular D_f_	Per SD decrease	− 0.23 (− 1.06 to 0.61)	0.594	− 0.28 (− 2.62 to 2.07)	0.816	− 0.11 (− 0.94 to 0.73)	0.804	− 0.17 (− 2.53 to 2.19)	0.887
Arteriolar tortuosity	Per SD decrease	− 0.43 (− 1.26 to 0.41)	0.312	− 1.00 (− 3.34 to 1.34)	0.399	− 0.45 (− 1.23 to − 0.33)	0.259	− 1.12 (− 3.35 to 1.12)	0.325
Venular tortuosity	Per SD decrease	− 0.14 (− 0.98 to 0.69)	0.734	− 0.204 (− 2.55 to 2.14)	0.863	− 0.35 (− 1.14 to 0.44)	0.384	− 0.70 (− 2.95 to 1.55)	0.538
Arteriolar Branching Angle	Per SD decrease	− 1.42 (− 2.22 to − 0.61)	0.001	− 3.50 (− 5.78 to − 1.23)	0.003	− 1.10 (− 1.90 to − 0.30)	0.007	− 2.85 (− 5.14 to − 0.57)	0.014
Venular branching angle	Per SD decrease	− 1.10 (− 1.92 to − 0.28)	0.009	− 3.05 (− 5.35 to − 0.76)	0.009	− 0.96 (− 1.73 to − 0.19)	0.015	− 2.67 (− 4.86 to − 0.48)	0.017
**OCT-A capillary metrics**									
cpVD	Per SD decrease	− 2.56 (− 3.29 to − 1.82)	< 0.001	− 7.06 (− 9.14 to − 4.99)	< 0.001	− 2.34 (− 3.11 to − 1.56)	< 0.001	− 6.46 (− 8.68 to − 4.25)	< 0.001
cpFD	Per SD decrease	− 2.67 (− 3.40 to − 1.95)	< 0.001	− 7.80 (− 9.81 to − 5.79)	< 0.001	− 2.48 (− 3.21 to − 1.75)	< 0.001	− 7.36 (− 9.41 to − 5.31)	< 0.001

*CI* confidence interval; *CRAE* central retinal arteriolar equivalent; *CRVE* central retinal venular equivalent; *cpVD* circumpapillary vessel density; *cpFD* circumpapillary fractal dimension; *D*_*f*_ fractal dimension; *MD* mean deviation; *SAP* standard automated perimetry; *SD* standard deviation; *VFI* visual field index.

Multivariable model adjusted for age, sex, axial length, systolic blood pressure, fellow caliber (for CRAE and CRVE), and OCT-A image quality score (for OCT-A capillary metrics).

A sensitivity analysis was also performed by excluding subjects with systemic cardiovascular diseases (hypertension, OSA, cerebrovascular accident and ischaemic heart disease) and subsequently determining the odds of NTG in relationship to decreased measurements in the microvascular metrics and the relationships between the microvascular metrics and glaucoma-related structural and functional changes (Supplementary Tables S2 to S4 online). The results were largely similar as those presented above (Tables 2, 3, 4).

Finally, we evaluated the strength of associations between retinal photographic arteriolar and venular and OCT-A capillary metrics with NTG and the glaucomatous changes in terms of average RNFL thickness, SAP MD and VFI (Supplementary Table S5 online). We sought to find out if significant differences exist between the regression models constructed using photographic arteriolar and venular metrics and OCT-A capillary metrics, respectively. We observed that logistic and liner regression models constructed using cpVD and cpFD both consistently showed stronger associations than those using photographic arteriolar and venular metrics for studying the associations with NTG and the glaucomatous changes (*P* < 0.05 in Z-test and Steiger’s Z-test).

## Discussion

In this study, our results revealed generalized attenuations in both photographic arteriolar and venular metrics and OCT-A capillary metrics (cpVD and cpFD) were associated with NTG and the structural (average RNFL thickness) and functional (SAP) glaucomatous measurements. We further demonstrated that OCT-A capillary metrics have stronger associations with NTG than that of photographic metrics.

Previously, studies reported attenuations in retinal microvasculature (arteriolar, venular and capillary networks) in POAG eyes compared with normal eyes^13–15,22^. However, inclusion of POAG eyes in which high-tension glaucoma constitute a vast majority, if not all, of the study subjects made it impossible to distinguish between worse microvasculature as a cause of ischemia resulting in RGC loss and glaucoma or in reverse elevated IOP causing structural damage and neural tissue loss in glaucoma to drive attenuations in microvasculature through decreased metabolic demands^29^. Studies in the past revealed a higher prevalence of disc hemorrhage in NTG eyes compared with other glaucoma eyes and the association between disc hemorrhage in NTG and systemic vascular risk factors^11,12,30^. These altogether suggest vascular factors may be of a determinant role in NTG pathogenesis compared to other forms of glaucoma and merits independent investigation. Our study therefore sought to understand the relationship between retinal microvasculature and this non-high-tension glaucoma subtype which has a long-standing hypothesis that microvascular dysfunction plays a key role in its pathogenesis in the absence of elevated IOP^9,10^. Our findings of the associations between microvascular attenuations and NTG are consistent with the widespread vascular alterations in the ONH, retina, choroid, and retrobulbar circulations in glaucoma^31–33^. This demonstrates the strong relationships between microvascular factors and NTG and supports the hypothesis of their potential roles in the pathogenic processes of NTG where a background of elevated IOP is absent. Further longitudinal studies to evaluate the temporal and causal relationships between generalized microvascular attenuations and NTG development or progression to ascertain such hypothesis are warranted.

In the past, the only easily available non-invasive measurement of retinal microvascular changes was confined to arterioles and venules measured from fundus photographs using computer-assisted software. This has been applied to large population-based studies to evaluate the vascular associations in POAG, such as the Blue Mountains Eye Study (n = 3654) and the Singapore Malay Eye Study (n = 2789)^13,15^. The summary findings in the current study and these previous studies were compared and outlined in Supplementary Table S6 online. Our findings in the associations of retinal photographic vascular metrics and NTG were in line with and corroborated the previous findings in POAG. Among the photographic vascular metrics, we found that arteriolar branching angle showed the most consistent associations with NTG throughout the different regression analyses. Although we do not have a definitive explanation for this observation, it is possible that the arterial branching pattern reflects the efficiency of retinal circulation and its attenuations (i.e. narrow branching angle) may reflect impaired perfusion, endothelial dysfunction and oxygen desaturation^34–36^. Previous studies showed that narrower arteriolar branching angle is associated with systemic vascular pathologies such as hypertension^37^. If microcirculatory insufficiency at all has a role in NTG pathogenesis, the arteriolar branching angle has potential utility as a sentinel microvascular marker for NTG. Further studies are warranted to evaluate the research and clinical utility of this parameter.

OCT-A now allows measurement of capillary network changes beyond retinal arteriolar and venular changes. A recent study using OCT-A reported that the removal of larger vessels (i.e., arterioles and venules) avoids the measurement of cpVD being confounded by the presence of beta-zone parapapillary atrophy (PPA) which may be present and increase with the severity of glaucoma^38^. In our study, retinal arterioles and venules on the original OCT-A images were hence removed to provide more accurate assessment of the capillary network in NTG eyes using OCT-A. We found that NTG eyes had decreased cpVD compared with normal subjects and decreased cpVD was associated with thinner RNFL, lower SAP MD and VFI. These are comprehensive evidences to support the associations of capillary network attenuations and NTG which corroborate with findings in previous OCT-A studies using relatively small samples of NTG patients (all n ≤ 31) in which similar findings were reported^25–28,39^.

Meanwhile, previous studies only focused on the use of cpVD to quantify the peripapillary capillary network. The fractal dimension (FD), a mathematical measure that quantifies complex geometric patterns in objects that are self-similar in their scaling patterns^40–42^, in the current context being retinal capillary vasculature, has not been quantified using OCT-A in previous studies on glaucoma nor applied to study NTG . The retinal FD is a measure of the complexity of the retinal vasculature and a reflection of its optimality and the efficiency of blood flow distribution with the least amount of energy^43^. Our results revealed that cpFD was significantly decreased in NTG as compared with normal eyes, and decreased cpFD was also associated with thinner RNFL, lower SAP MD and VFI. This further supported an association between microcirculatory dysfunction indicated by worse cpFD and NTG, and suggested the possible utility of cpFD in future NTG microvasculature assessments. All in all, these findings utilizing OCT-A capillary metrics offered evidence to support the relationships between capillary rarefaction and NTG, and the possible role of microcirculatory insufficiency in the pathogenesis of NTG as hypothesized in the vascular theory of glaucoma^32,44^.

In this study, we also provided the first evidence to demonstrate that the associations of NTG and glaucoma-related structural and functional changes with OCT-A capillary metrics were stronger and more consistent than that with photographic arteriolar and venular metrics. It is thought that vascular dysfunction and hypoxia lead to oxidative injury of retinal ganglion cells and contribute to the pathogenesis of glaucoma^45,46^. Previous in-vitro studies have shown that formation of peroxynitrite (OONO-) and increased expression of vascular endothelial growth factor (VEGF) in the hypoxic retina resulted in increased retinal vascular permeability and disruption of the blood-retina barrier (BRB)^47,48^. The dysfunctional BRB with hyperpermeability and increased expression of monocyte chemoattractant protein-1 (MCP-1) in retinal hypoxia–ischemia attract monocytes from circulation and resident microglia to infiltrate hypoxic areas^49^, resulting in their activation and the subsequent inflammatory response^50,51^. The hypoxia-activated macrophages and microglia release proinflammatory molecules such as tumor necrosis factor-α (TNF-α), interleukin-1ß (IL-1ß), inducible nitric oxide synthase (iNOS), intercellular cell adhesion molecule-1 (ICAM-1) and cyclooxygenase-2 (COX-2)^52,53^. These inflammatory molecules released in such state of neuroinflammation are known play a critical role in degeneration of retinal capillaries and RGC death^54–56^. Peripapillary capillaries were observed to atrophy proportionally with retinal nerve fibers in glaucoma^57^. These evidences support the intimate relationship between concomitant degeneration of retinal capillary microvasculature and RGC death in the complex pathologic processes with retinal hypoxia–ischemia ultimately culminating in glaucoma. Although current evidence is insufficient to indicate a direct causal relationship between capillary vasculature atrophy and RGC degeneration, degeneration in capillary vasculature is theoretically an ideal surrogate marker of vascular dysfunction, RGC degeneration and glaucoma. Our findings in this study with NTG patients lend support to such hypothesis by demonstrating the strong and consistent associations of NTG and the underlying glaucomatous changes with OCT-A capillary metrics. OCT-A capillary metrics may therefore be of utility in future studies of NTG vasculature and can potentially be assimilated into the standard of care to aid diagnosis and monitoring of NTG progression given their strong associations^58^.

The strength of our study is the inclusion of only NTG patients instead of any types of POAG patients, which should better reflect and support the hypothesis of the role of vascular factors in the pathogenesis of glaucoma in the absence of elevated IOP. Furthermore, the current study presents a comprehensive assessment of both retinal photographic arteriolar and venular and OCT-A capillary metrics in NTG. To our knowledge, this is the first glaucoma study documenting assessments in both sets of vascular metrics and their associations with both structural and functional glaucomatous changes. Limitations should also be noted. Firstly, because of the cross-sectional design, we could only report the associations of generalized microvascular metrics attenuations with NTG but unable to establish any causal relationships. The microvasculature atrophy could either be a triggering event of retinal ischemia, RGC loss and NTG development or as a direct consequence driven by reduced metabolic demands following structural and functional degeneration in NTG. The results should be interpreted in consideration of both scenarios. Longitudinal studies are therefore warranted to evaluate the temporal relationships between microvascular metrics attenuations and NTG development and progression as reflected by structural and functional glaucomatous measurements to ascertain the underlying causal relationships. Secondly, the use of retinal fundus photographs and SIVA as a semi-automated computer-assisted software for evaluation of retinal vasculature was operator-dependent and human variability were inevitable in the estimation of the photographic vascular metrics. In addition, the measured areas between retinal photographs used for generating arteriolar and venular metrics and OCT-A images used for generating capillary metrics were different, which may account for the discrepancies between the strength of associations of the two sets of vascular metrics with NTG and the underlying glaucomatous changes. This however reflects the intrinsic nature of the two imaging modalities in reality settings, hence their respective efficacy and accuracy in NTG microvasculature assessment. Finally, our study population comprised of only subjects of Chinese ethnicity, which may limit the generalizability of our findings. Nonetheless, since NTG represents the majority of POAG cases in Asia, these findings are best suited for clinical practice and future NTG studies in which patients of such ethnicity would likely constitute a significant majority.

In conclusion, we have demonstrated generalized changes in retinal arteriolar, venular and capillary metrics in NTG eyes. We further demonstrated that OCT-A capillary metrics showed stronger associations with NTG than fundus photographic arteriolar and venular metrics. These findings provide additional insights into the role of vascular factors in NTG and the potential utility of OCT-A capillary metrics in NTG assessments, diagnostics and progression monitoring, given its strong associations with NTG and the underlying structural and functional glaucomatous changes.

## Methods

### Study population

This is a clinic based, case–control study. A total of 115 patients with NTG and 70 normal subjects were consecutively recruited from the Hong Kong Eye Hospital and the CUHK Eye Center of The Chinese University of Hong Kong from 2016 to 2018. For inclusion in this study, participants have to be diagnosed with NTG according to the definition below and of 18 years of age or above. Control subjects had to meet their respective criteria as listed below. One eye from each subject was selected for the current analysis. All participants had visual acuity of 20/40 or better. Subjects with diabetes mellitus, maculopathy, other optic neuropathies, history of ocular surgery (except cataract surgery), neurologic disease, or major systemic illness were excluded. This study was conducted in accordance with the 1964 Declaration of Helsinki and was approved by the Research Ethics Committee (Kowloon Central / Kowloon East) of Hospital Authority (Ref: KC/KE-17-0099/ER-3). Written informed consent was obtained from all subjects.

### Clinical examination

All participants underwent a comprehensive ophthalmologic examination at baseline, including measurements of best-corrected visual acuity, refractive error by an autorefractor (ARK-510A, Nidek Co., Ltd., Gamagori, Japan), axial length (IOL Master, Carl Zeiss Meditec, Dublin, US), intraocular pressure (IOP) by Goldmann applanation tonometry, central corneal thickness (CCT) by a noncontact tonopachymeter (TONOPACHY 530P, Nidek Co., Ltd., Gamagori, Japan), slit-lamp biomicroscopy examination of the optic disc and retina, dilated fundus examination, simultaneous stereophotography of the optic disc, standard automated perimetry (SAP, Humphrey Field Analyzer; 24-2 Swedish interactive threshold algorithm; Carl Zeiss Meditec Inc., Dublin, CA, USA) were performed. Peripapillary retinal nerve fiber layer (RNFL) thickness was measured with Cirrus HD-OCT (Carl Zeiss Meditec Inc., Dublin, CA, USA). Systolic and diastolic blood pressure (SBP and DBP) and pulse rate were measured thrice with an Omron automatic blood pressure instrument (Omron Avant 2120; Nonin Medical, Inc., Plymouth, MN, USA), and the mean values were used in the analysis.

### Definition of NTG

NTG was diagnosed based on: (1) Six median untreated IOP readings consistently less than 21 mmHg, with no more than 1 reading equal to 23 or 24 mmHg, and no single measurement more than 24 mmHg, as per the Collaborative NTG Study^59^. At least 2 readings were obtained at different times of the day from the rest. (2) Drainage angle of Shaffer grade II or above on dark room gonioscopy. (3) Glaucomatous optic disc cupping and loss of neuroretinal rim. (4) Visual field defect according to the Collaborative NTG Study^59^. (5) Glaucoma hemifield test being ‘outside normal limits’. (6) Pattern standard deviation with a *P* value < 0.05. (7) A cluster of 3 points or more in the pattern deviation plot in a single hemifield with a *P* value < 0.05, one of which must have a *P* value < 0.01. Any one of these criteria, if repeatable, was considered to be sufficient evidence of a glaucomatous VF defect. (8) Absence of secondary causes for the glaucomatous optic neuropathy (e.g. previous trauma, use of steroids, and uveitis, etc.). Normal controls had anterior chamber angle of Shaffer grade II or above, no optic disc abnormalities in clinical examination, no visual field abnormalities on visual field testing, and no family or personal history of glaucoma, or other ocular diseases except visually insignificant senile cataract, or myopia or hyperopia of less than 3 diopters.

### Standard automated perimetry

All participants underwent visual field testing using the 24-2 pattern Swedish interactive threshold algorithm (SITA) on the Humphrey Field Analyzer (Carl Zeiss Meditec Inc., Dublin, CA, USA) within 6 months of imaging. Only reliable tests (≤ 33% fixation losses and false- negatives, and ≤ 15% false-positives) were included^60^. The quality of visual field tests was also reviewed by study investigators to identify and exclude visual fields with evidence of inattention or inappropriate fixation, artefacts such as eyelid and lens rim artefacts, fatigue effects, and abnormal results caused by diseases other than glaucoma.

### Quantitative Retinal arteriolar and venular analysis with fundus photography

**Figure 1:**
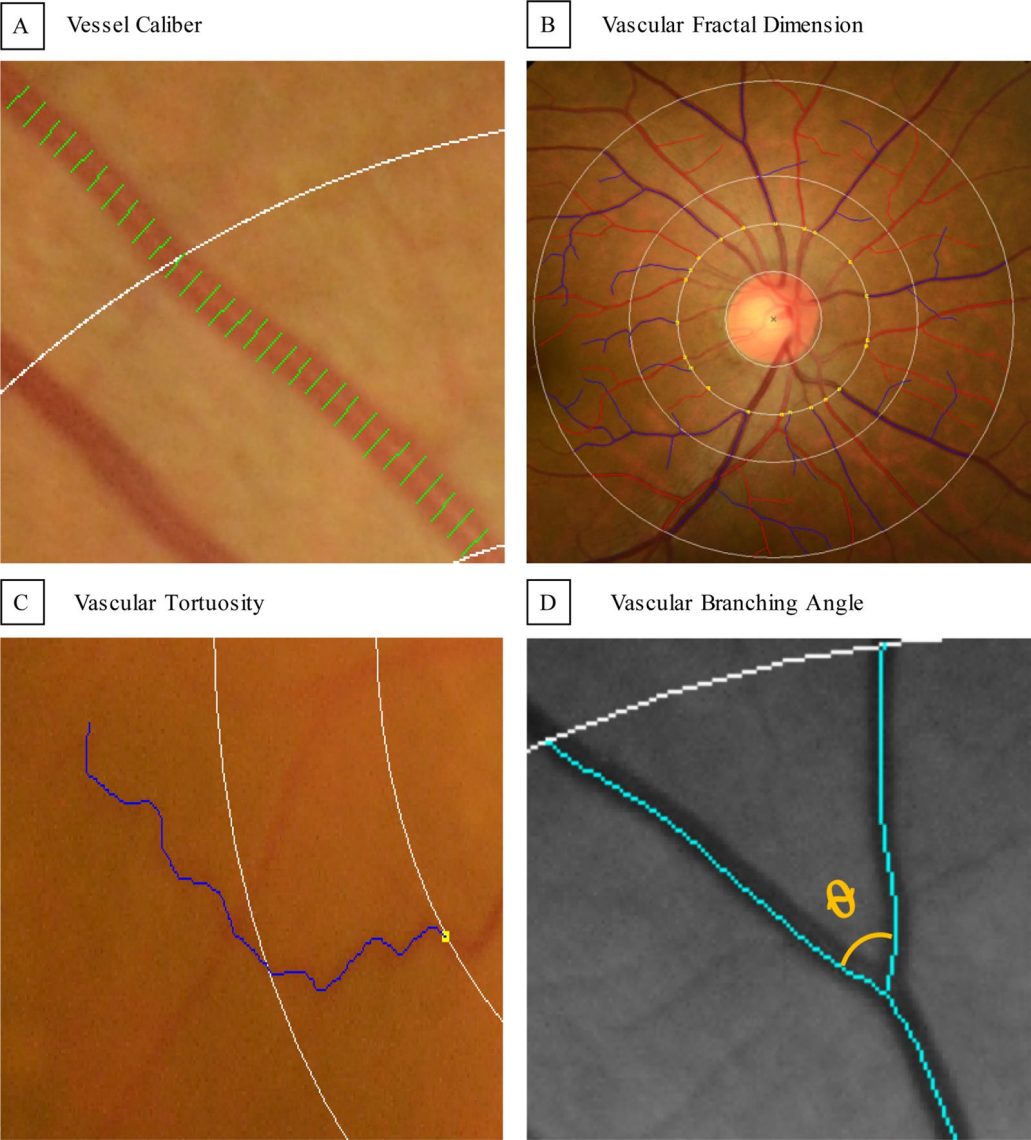
Quantification of retinal photographic arteriolar and venular metrics. Measurement of retinal photographic vascular metrics are generated from retinal photographs by a semi-automated computer-assisted program (Singapore I Vessel Assessment [SIVA], version 4.0, National University of Singapore, Singapore. https://retinaresearch.d2.comp.nus.edu.sg/users/sign_in). (**A**) Vessel covers are measurement lines used to estimate the retinal vessel calibers. Shown in the image are vessel covers laid on an arteriole for estimation of its caliber by the SIVA program. (**B**) All vessels within zones B and C were traced for calculation of fractal dimension by the SIVA program using the ‘box-counting’ method. (**C**) A tortuous venule traced and measured by the SIVA program. Retinal vascular tortuosity was defined as the integral of the curvature square along the path of the vessel, normalized by the total path length. (**D**) A branching angle θ as defined by the first angle subtended between 2 daughter vessels at each vascular bifurcation measured by the SIVA program.

Disc-centered digital retinal fundus photographs were obtained from all participants using a non-mydriatic retinal camera (TRC 50DX, Topcon Inc., Tokyo, Japan) after pharmacological pupil dilation. A series of retinal photographic vascular metrics (vessel caliber, vascular fractal dimension, vascular tortuosity and vascular branching angle) were measured by a semi-automated computer-assisted program (Singapore I Vessel Assessment [SIVA], version 4.0, National University of Singapore, Singapore) from the disc-centered retinal photographs (ETDRS field 1)^61^, according to a standardized grading protocol by three trained graders (TPL, WYM, KH) who were masked to the study subjects applied SIVA to each image (Fig. 1). Details of the measurement were available in the Supplementary Methods online and the intergrader reliability was reported in Supplementary Table S7 online.

### OCT-A imaging

Prior to OCT-A imaging, pupils were dilated with tropicamide and phenylephrine (0.5% each). All participants then underwent OCT-A with a swept-source OCT (Triton DRI-OCT, Topcon, Inc, Tokyo, Japan), which contained a swept-source with a wavelength of 1,050 nm light source and a speed of 100,000 A-scans per second. A volumetric OCT scan centred on the optic nerve head was obtained from each eye, with a scan area of 3 mm by 3 mm containing 320 × 320 A-scans for each volumetric scan. An image quality score ranging from 0 to 100 was given by the software for each volumetric OCT scan. We used the built-in software (IMAGEnet6) to generate horizontal depth-resolved OCT-A slabs to provide improved detection sensitivity of low blood flow and reduced motion artefacts without compromising axial resolution^62^. In the current analysis, we selected radial peripapillary capillary network from the ONH scan which comprises of capillary beds within RNFL layer predominantly.

### OCT-A quality control

Two readers (YMW, TPL) carefully evaluated each OCT-A image in the CUHK Ocular Reading Centre. The readers were masked to all study subjects. OCT-A images with significant image artefacts and poor image quality were excluded from analysis, including (1) quality score of below 40; (2) motion artefacts (e.g. vessel discontinuity or significant residual motion lines); (3) inaccurate segmentation of tissue layers or slabs; (4) blurry images (e.g. due to axial movement or media opacity); (5) signal loss (e.g. due to eye blinking) or (6) poor centration (i.e. ONH not at center). All the OCT-A scans met the image quality requirement for inclusion in the current analysis.

### Quantitative OCT-A metrics

**Figure 2:**
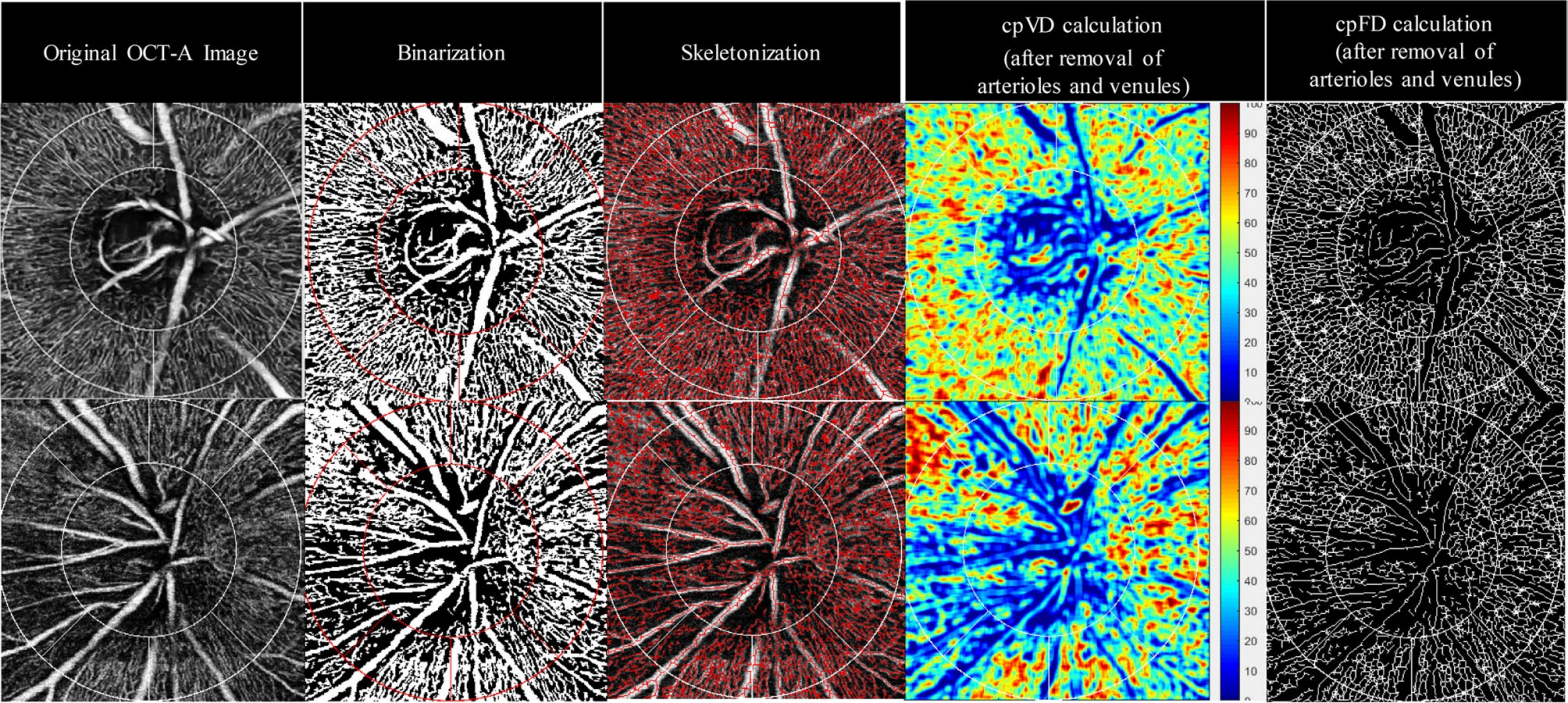
Quantification of OCT-A Capillary Metrics in Normal and NTG eyes. Measurement of OCT-A capillary metrics are generated from ONH OCT-A images by a semi-automated customized MATLAB program (MATLAB R2017a, The MathWorks, Natick, MA, USA. https://www.mathworks.com/products/matlab.html). (From left to right) ONH OCT-A images with the cpVD and cpFD measurement region defined. Binarization of the ONH OCT-A images with white pixels representing the blood vessels and black pixels representing the background (non-perfusion regions). Skeletonization of the ONH OCT-A images. Analysis and quantification of cpVD after removal of retinal arterioles and venules from the binarized ONH OCT-A images. Analysis and quantification of cpFD after removal of retinal arterioles and venules from the skeletonized ONH-OCT-A images. (Top: an example of normal eye. Bottom: an example of NTG eye).

The OCT-A images were exported in grayscale from the built-in software and imported into a semi-automated customized MATLAB program (MATLAB R2017a, MathWorks, Natick, MA, USA) for image analysis. The circumpapillary vessel density (cpVD) and circumpapillary fractal dimension (cpFD) within a region defined as a circular annulus with 3 mm outer diameter and inner boundary fitted to the optic disc based on 360° global area were analysed (Fig. 2). The inter- and intra-grader reliability were high for both measurements, with intraclass correlation coefficients (ICC) ranging from 0.943 to 0.998 (Supplementary Table S8 online).

A non-local means (NLM) denoising filter was first applied to the grayscale images in order to reduce the background noise, sharpen the blood vessels and improve signal-to-noise ratio while preserving the image features after the OCT-A images were exported in grayscale and imported to an automated customized MATLAB program^63^. The NLM filter is a non-linear neighborhood filter in which the pixel/voxel value to be restored is replaced by a weighted average of the pixel intensities in the entire noisy image. The denoised image was then binarized, using phansalkar adaptive local thresholding method with white pixels representing the blood vessels and black pixels representing the background (non-perfusion regions), or skeletonized^64^, respectively.

Retinal arterioles and venules were removed before subsequent analyses on circumpapillary vessel density (cpVD) and fractal dimension (cpFD) were conducted. The detection of the retinal vessels was based on the original OCT-A image extracted from RPC layer (defined by built-in software of Triton OCT). The vessel removal mask was generated by combining masks processed from two different approaches. For the first approach, the RPC layer image was denoised with averaging filter and median filter. The denoised image was subsequently enhanced with contrast stretching. Part of the retinal vessels was then extracted by binarization with global thresholding on the contrast-stretched image with reference to the overall brightness of the image. For the second approach, the RPC layer image was first processed with contrast-limited adaptive histogram equalization to adaptively enhance the regional contrast. The processed image was subsequently denoised with averaging filter and median filter, and further enhanced with contrast stretching. Another part of the retinal vessels was then extracted by binarization with 2-D Frangi filter. Both the first mask and the second mask were further processed with morphological opesrations to remove artefacts. A final mask representing all retinal vessels (arterioles and venules) was computed by union of the two detected masks and applied to the original grayscale OCT-A image for vessel removal.

cpVD was calculated as the percentage of area not defined as non-perfusion regions over the total area in the region defined above from the binarized ONH OCT-A image, after removal of the retinal arterioles and venules. cpFD was calculated from the skeletonized^64^ ONH OCT-A image using “box-counting method”^65,66^ in the same region defined above, after removal of the retinal arterioles and venules.

### Structural OCT imaging

Cirrus HD-OCT (software version 9.5; Carl Zeiss Meditec, Dublin, CA, USA) was used to measure the average RNFL thicknesses from the optic disc cube scan (200 × 200 pixels), generating the RNFL thickness map at the peripapillary region (6 mm × 6 mm). Average RNFL thickness was used for evaluation of the associations with photographic arteriolar and venular and OCT-A capillary metrics. All OCT scans had signal strength of 6 or above.

### Statistical analysis

Statistical analysis was performed using SPSS version 25 (SPSS, Inc., Chicago, IL, USA). Photographic arteriolar and venular metrics (vessel caliber, fractal dimension, tortuosity and branching angle) generated from SIVA and OCT-A capillary metrics (cpVD and cpFD) were analyzed as continuous variables. Clinical characteristics, photographic arteriolar and venular metrics and OCT-A capillary metrics were compared between NTG patients and normal controls. For continuous variables, independent sample *t* test was used. For categorical variables, the chi-square test was performed. Logistic regression models were constructed to determine odds ratios (ORs) and 95% confidence intervals of NTG in relation to each standard deviation change in the microvascular imaging metrics, adjusting for age, sex and axial length (AL), systolic blood pressure (SBP), fellow caliber (for vessel caliber-related analyses) and OCT-A quality score (for OCT-A capillary metrics-related analyses). Multivariable linear regression models adjusting for the aforementioned variables were used to estimate changes in average RNFL thickness (also adjusting for OCT signal strength), MD and VFI for each standard deviation change in the microvascular metrics. Finally, the different regression models were compared to determine if photographic arteriolar and venular or OCT-A capillary metrics reflect stronger associations with NTG and the glaucomatous structural and functional alterations using Z-test for comparison of logistic regression models and Steiger’s Z-tests for comparison of linear regression models.

## Supplementary information

Supplementary Information

## Data Availability

All data generated or analysed during this study are included in this published article (and its Supplementary Information files).
