# Supplementary material for: Global assessment of arteriolar, venular and capillary changes in normal tension glaucoma
Source: Sci Rep. 2020 Nov 5;10:19222. doi: 10.1038/s41598-020-75784-1 (PMC7644687; doi:10.1038/s41598-020-75784-1)
Supplement: Supplementary file 1 — Supplementary Information [file 41598_2020_75784_MOESM1_ESM.pdf]

Supplementary Information

Global Assessment of Arteriolar, Venular and Capillary Changes in Normal Tension Glaucoma

Timothy P. H. Lin;[1‡] Yu Meng Wang;[1‡] Kevin Ho;[1] Cherie Y. K. Wong;[1,2] Poemen P. Chan;[1,2] Mandy O. M. Wong;[1,2] Noel C. Y. Chan;[1,3] Fangyao Tang;[1] Alexander Lam;[1] Dexter Y. L. Leung;[1,4] Tien Yin Wong;[5] Ching-Yu Cheng;[5] Carol Y. Cheung;[1†*] Clement C. Tham[1,2†]

1. Department of Ophthalmology and Visual Sciences, The Chinese University of Hong Kong, Hong Kong
2. Hong Kong Eye Hospital, Hong Kong
3. Department of Ophthalmology, Prince of Wales Hospital, Hong Kong
4. Department of Ophthalmology, Hong Kong Sanatorium and Hospital, Hong Kong
5. Singapore Eye Research Institute, Singapore National Eye Center, Singapore

‡These authors contributed equally to the work as co-first authors.

† These authors contributed equally to the work as senior authors.

***Corresponding author:**

Dr. Carol Y. Cheung

Telephone: (852) 3943 0791

Email: carolcheung@cuhk.edu.hk

Supplementary Methods

Quantitative Retinal Vascular Metrics Analysis with Fundus Photography

In measurement of retinal photographic arteriolar and venular metrics, the Singapore I Vessel Assessment (SIVA) (version 4.0, National University of Singapore, Singapore) was applied to each disc-centered retinal photograph by one of the three trained graders (TPL, YMW, KH) who was masked to the image.

A grid, after appropriately identifying the center of the optic disc, divided the image into 3 concentric zones [zone A (within 0.5 disc diameter away from the optic disc margin), B (0.5 and 1 disc diameter away from the optic disc margin), and C (0.5 and 2 disc diameters away from the optic disc margin)]. All vessels within zones B and C were traced and identified as either arterioles or venules. The software has an automated function to appropriately identify arterioles and venules, indicated by 2 different colored lines generated by the program, red for arterioles and blue for venules. The graders subsequently checked each image to confirm if all arterioles and venules were correctly identified, based on information of parent vessels, crossing between arterioles and venules, and the color of the vessels. Corrections were made when necessary.

- For assessment of vessel caliber, the biggest 6 arterioles and venules coursing through zones B and C were traced and the vessel calibers of these vessels were measured using a widely-adopted method modified from the ARIC study.[1,2] This involved measuring the length of the vessel covers in the six largest arterioles and venules respectively to estimate their vessel calibers (**Figure 1a**) and combining such

measurements into summary measures, which were referred to as central retinal arteriolar equivalent (CRAE) and central retinal venular equivalent (CRVE) using the revised Parr-Hubbard formulas as described by Knudtson et al.[3,4]

- Retinal vascular fractal dimension was calculated from a skeletonized line tracing of the retinal vascular bed, using the established “box-counting method”, as a global measure that summarizes the complexity and whole branching pattern of the retinal vascular tree (**Figure 1b**). A larger value indicates a more complex branching pattern.[5-7]
- Retinal vascular tortuosity was defined as the integral of the curvature square along the path of the vessel, normalized by the total path length (**Figure 1c**). A smaller tortuosity value indicates a straighter vessel. The estimates were summarized as retinal arteriolar tortuosity and retinal venular tortuosity, representing respectively the average tortuosity of arterioles and venules of the eye.[8,9]
- Retinal vascular branching angle was defined as the first angle subtended between 2 daughter vessels at each vascular bifurcation (θ) (**Figure 1d**). The estimates were summarized as retinal arteriolar branching angle and retinal venular branching angle, representing respectively the average branching angle of arterioles and venules of the eye.[10]

Supplementary Table S1. Comparison of Retinal Photographic and OCT-A Microvascular Metrics between NTG and Controls

**Retinal Microvascular Metrics**	**Controls (n=68)**	**NTG (n=100)**	***P* value**
**Photographic Vascular Metrices**			
CRAE (μm)	**158.28 (15.21)**	**151.21 (14.57)**	**0.003**
CRVE (μm)	**225.08 (20.29)**	**213.96 (22.08)**	**0.001**
Arteriolar D_f	1.22 (0.05)	1.21 (0.06)	0.204
Venular D_f	1.21 (0.04)	1.21 (0.05)	0.63
Arteriolar Tortuosity (x 10^4)	**0.67 (0.16)**	**0.61 (0.17)**	**0.03**
Venular Tortuosity (x 10^4)	0.74 (0.19)	0.70 (0.20)	0.154
Arteriolar Branching Angle (°)	**80.20 (13.31)**	**73.42 (12.98)**	**0.001**
Venular Branching Angle (°)	**80.14 (8.97)**	**77.32 (9.19)**	**0.05**
**OCT-A Capillary Metrics**			
cpVD (%)	**56.0 (5.00)**	**51.0 (7.20)**	**<0.001**
cpFD	**1.548 (0.021)**	**1.513 (0.038)**	**<0.001**

CRAE = central retinal arteriolar equivalent; CRVE = central retinal venular equivalent; cpVD = circumpapillary vessel density; cpFD = circumpapillary fractal dimension; D_f = fractal dimension; NTG = normal tension glaucoma.

Supplementary Table S2. Relationship of Retinal Photographic and OCT-A Microvascular Metrics with NTG

**Retinal Microvascular Metrics**		**NTG, OR (95% CI)**	***P* Value**
**Photographic Vascular Metrics**			
CRAE	per SD decrease	0.99 (0.49-2.00)	0.97
CRVE	per SD decrease	1.34 (0.68-2.67)	0.401
Arteriolar D_f	per SD decrease	0.87 (0.53-1.41)	0.567
Venular D_f	per SD decrease	1.09 (0.69-1.74)	0.709
Arteriolar Tortuosity	per SD decrease	**1.74 (1.08-2.81)**	**0.024**
Venular Tortuosity	per SD decrease	**1.90 (1.11-3.24)**	**0.019**
Arteriolar Branching Angle	per SD decrease	**1.78 (1.07-2.94)**	**0.025**
Venular Branching Angle	per SD decrease	1.46 (0.91-2.33)	0.113
**OCT-A Capillary Metrics**			
cpVD	per SD decrease	**2.92 (1.49-5.76)**	**0.002**
cpFD	per SD decrease	**20.67 (5.65-75.58)**	**<0.001**

CI = confidence interval; CRAE = central retinal arteriolar equivalent; CRVE = central retinal venular equivalent; cpVD = circumpapillary vessel density; cpFD = circumpapillary fractal dimension; D_f = fractal dimension; NTG = normal tension glaucoma; OR = odds ratio; SD = standard deviation.
Adjusted for age, sex, axial length, systolic blood pressure, fellow caliber (for CRAE and CRVE) and OCT-A image quality score (for OCT-A capillary metrics).

Supplementary Table S3. Relationship of Retinal Photographic and OCT-A Microvascular Metrics with Average RNFL Thickness

**Retinal Microvascular Metrics**		**Average RNFL Thickness, μm (95% CI)**	***P* Value**
**Photographic Vascular Metrics**			
CRAE (also adjusted for CRVE)	per SD decrease	-1.80 (-6.52 to 2.91)	0.449
CRVE (also adjusted for CRAE)	per SD decrease	-2.56 (-7.02 to 1.91)	0.259
Arteriolar D_f	per SD decrease	0.32 (-2.94 to 3.58)	0.845
Venular D_f	per SD decrease	-0.19 (-3.27 to 2.88)	0.902
Arteriolar Tortuosity	per SD decrease	-1.36 (-4.10 to 1.39)	0.329
Venular Tortuosity	per SD decrease	-1.27 (-4.43 to 1.90)	0.429
Arteriolar Branching Angle	per SD decrease	**-2.76 (-5.49 to -0.26)**	**0.048**
Venular Branching Angle	per SD decrease	**-5.00 (-7.79 to -2.22)**	**0.001**
**OCT-A Capillary Metrics**			
cpVD	per SD decrease	**-6.71 (-9.75 to -3.68)**	**<0.001**
cpFD	per SD decrease	**-8.17 (-11.10 to -5.25)**	**<0.001**

CI = confidence interval; CRAE = central retinal arteriolar equivalent; CRVE = central retinal venular equivalent; cpVD = circumpapillary vessel density; cpFD = circumpapillary fractal dimension; D_f = fractal dimension; NTG = normal tension glaucoma; OR = odds ratio; RNFL = retinal nerve fiber layer; SD = standard deviation. Adjusted for age, sex, axial length, systolic blood pressure, fellow caliber (for CRAE and CRVE), OCT signal strength and OCT-A image quality score (for OCT-A capillary metrics).

Supplementary Table S4. Relationship of Retinal Photographic and OCT-A Microvascular Metrics with Standard Automated Perimetry Measurements

**Retinal Microvascular Metrics**		**SAP MD, dB (95% CI)**	***P* Value**	**SAP VFI, % (95% CI)**	***P* Value**
**Photographic Vascular Metrics**					
CRAE	per SD decrease	-0.34 (-2.12 to 1.44)	0.704	-2.34 (-7.26 to 2.59)	0.348
CRVE	per SD decrease	-0.60 (-2.32 to 1.13)	0.492	-0.55 (-5.33 to -4.23)	0.82
Arteriolar D_f	per SD decrease	-1.00 (-2.23 to 0.24)	0.113	-2.66 (-6.09 to 0.78)	0.128
Venular D_f	per SD decrease	0.30 (-0.87 to 1.46)	0.612	1.27 (-1.96 to 4.50)	0.436
Arteriolar Tortuosity	per SD decrease	-0.43 (-1.49 to 0.63)	0.422	-0.77 (-3.72 to 2.18)	0.605
Venular Tortuosity	per SD decrease	**-1.31 (-2.34 to -0.28)**	**0.014**	0.04 (-3.36 to 3.44)	0.981
Arteriolar Branching Angle	per SD decrease	-0.35 (-1.57 to 0.87)	0.572	**-3.19 (-6.09 to -0.30)**	**0.031**
Venular Branching Angle	per SD decrease	**-1.22 (-2.34 to -0.10)**	**0.033**	-3.12 (-6.23 to -0.003)	0.05
**OCT-A Capillary Metrics**					
cpVD	per SD decrease	**-3.52 (-4.59 to -2.44)**	**<0.001**	**-9.68 (-12.68 to -6.67)**	**<0.001**
cpFD	per SD decrease	**-3.44 (-4.54 to -2.33)**	**<0.001**	**-9.27 (-12.37 to -6.17)**	**<0.001**

CI = confidence interval; CRAE = central retinal arteriolar equivalent; CRVE = central retinal venular equivalent; cpVD = circumpapillary vessel density; cpFD = circumpapillary fractal dimension; D_f = fractal dimension; MD = mean deviation; SAP = standard automated perimetry; SD = standard deviation; VFI = Visual Field Index. Adjusted for age, sex, axial length, systolic blood pressure, fellow caliber (for CRAE and CRVE), and OCT-A image quality score (for OCT-A capillary metrics).

Supplementary Table S5. Multiple Logistic and Linear Regression Analyses of NTG or Glaucomatous Structural and Functional Changes in NTG (Dependent Variables) and Retinal Microvascular Metrics (Independent Variables)

**Retinal Microvascular Metrics**	**NTG**	**Average RNFL thickness, μm**		**SAP MD, dB**		**SAP VFI, %**	
	**OR (95% CI)**	**ß (95% CI)**	**R^2**	**ß (95% CI)**	**R^2**	**ß (95% CI)**	**R^2**
cpVD	2.77 (1.64-4.69)**	-4.95 (-7.05 to -2.85)**	0.326	-2.34 (-3.11 to -1.56)**	0.309	-6.46 (-8.68 to -4.25)**	0.278
CRAE	0.86 (0.49-1.53)	-0.44 (-3.68 to 2.80)	0.259	-0.30 (-1.53 to 0.92)	0.159	-1.93 (-5.41 to 1.55)	0.139
P-value†	**0.003**	0.179		**0.003**		**0.006**	
cpVD	2.77 (1.64-4.69)**	-4.95 (-7.05 to -2.85)**	0.326	-2.34 (-3.11 to -1.56)**	0.309	-6.46 (-8.68 to -4.25)**	0.278
CRVE	1.47 (0.85-2.56)	-3.22 (-6.29 to -0.14)*	0.259	-0.23 (-1.41 to 0.96)	0.159	0.14 (-3.22 to 3.50)	0.139
P-value†	**0.103**	0.179		**0.003**		**0.006**	
cpVD	2.77 (1.64-4.69)**	-4.95 (-7.05 to -2.85)**	0.326	-2.34 (-3.11 to -1.56)**	0.309	-6.46 (-8.68 to -4.25)**	0.278
Arteriolar D_f	0.95 (0.62-1.44)	0.31 (-2.11 to 2.73)	0.215	-0.85 (-1.76 to 0.50)	0.171	-2.40 (-4.96 to 0.17)	0.146
P-value†	**0.002**	**0.008**		**0.006**		**0.008**	
cpVD	2.77 (1.64-4.69)**	-4.95 (-7.05 to -2.85)**	0.326	-2.34 (-3.11 to -1.56)**	0.309	-6.46 (-8.68 to -4.25)**	0.278
Venular D_f	1.04 (0.70-1.53)	-0.82 (-3.06 to 1.41)	0.218	-0.11 (-0.94 to 0.73)	0.153	-0.17 (-2.53 to 2.19)	0.128
P-value†	**0.003**	**0.009**		**0.001**		**0.002**	
cpVD	2.77 (1.64-4.69)**	-4.95 (-7.05 to -2.85)**	0.326	-2.34 (-3.11 to -1.56)**	0.309	-6.46 (-8.68 to -4.25)**	0.278
Arteriolar Tortuosity	1.71 (1.15-2.52)*	-1.02 (-3.10 to 1.06)	0.22	-0.45 (-1.23 to -0.33)	0.16	-1.12 (-3.35 to 1.12)	0.134
P-value†	0.149	**0.01**		**0.002**		**0.002**	
cpVD	2.77 (1.64-4.69)**	-4.95 (-7.05 to -2.85)**	0.326	-2.34 (-3.11 to -1.56)**	0.309	-6.46 (-8.68 to -4.25)**	0.278
Venular Tortuosity	1.73 (1.15-2.59)*	-0.01 (-2.11 to 2.10)	0.215	-0.35 (-1.14 to 0.44)	0.157	-0.70 (-2.95 to 1.55)	0.13
P-value†	0.165	**0.008**		**0.002**		**0.002**	
cpVD	2.77 (1.64-4.69)**	-4.95 (-7.05 to -2.85)**	0.326	-2.34 (-3.11 to -1.56)**	0.309	-6.46 (-8.68 to -4.25)**	0.278
Arteriolar Branching Angle	1.52 (1.01-2.30)*	-2.78 (-4.90 to -0.65)*	0.246	-1.10 (-1.90 to -0.30)*	0.1	-2.85 (-5.14 to -0.57)*	0.16
P-value†	0.078	0.063		**0.019**		**0.018**	
cpVD	2.77 (1.64-4.69)**	-4.95 (-7.05 to -2.85)**	0.326	-2.34 (-3.11 to -1.56)**	0.309	-6.46 (-8.68 to -4.25)**	0.278
Venular Branching Angle	1.39 (0.96-2.01)	-2.69 (-4.72 to -0.66)*	0.247	0.176 (0.021 to 0.188)*	0.183	-2.67 (-4.86 to -0.48)*	0.158
P-value†	**0.035**	0.091		**0.019**		**0.025**	
cpFD	8.80 (3.93-19.70)**	-5.52 (-7.51 to -3.54)**	0.337	-2.48 (-3.21 to -1.75)**	0.342	-7.36 (-9.41 to -5.31)**	0.337
CRAE	0.86 (0.49-1.53)	-0.44 (-3.68 to 2.80)	0.259	-0.30 (-1.53 to 0.92)	0.159	-1.93 (-5.41 to 1.55)	0.139
P-value†	**<0.001**	0.069		**<0.001**		**<0.001**	
cpFD	8.80 (3.93-19.70)**	-5.52 (-7.51 to -3.54)**	0.337	-2.48 (-3.21 to -1.75)**	0.342	-7.36 (-9.41 to -5.31)**	0.337

CRVE	1.47 (0.85-2.56)	-3.22 (-6.29 to -0.14)*	0.259	-0.23 (-1.41 to 0.96)	0.159	0.14 (-3.22 to 3.50)	0.139
P-value†	**<0.001**	0.069		**<0.001**		**<0.001**	
cpFD	8.80 (3.93-19.70)**	-5.52 (-7.51 to -3.54)**	0.337	-2.48 (-3.21 to -1.75)**	0.342	-7.36 (-9.41 to -5.31)**	0.337
Arteriolar D_f	0.95 (0.62-1.44)	0.31 (-2.11 to 2.73)	0.215	-0.85 (-1.76 to 0.50)	0.171	-2.40 (-4.96 to 0.17)	0.146
P-value†	**<0.001**	**0.002**		**0.002**		**0.001**	
cpFD	8.80 (3.93-19.70)**	-5.52 (-7.51 to -3.54)**	0.337	-2.48 (-3.21 to -1.75)**	0.342	-7.36 (-9.41 to -5.31)**	0.337
Venular D_f	1.04 (0.70-1.53)	-0.82 (-3.06 to 1.41)	0.218	-0.11 (-0.94 to 0.73)	0.153	-0.17 (-2.53 to 2.19)	0.128
P-value†	**<0.001**	**0.003**		**<0.001**		**<0.001**	
cpFD	8.80 (3.93-19.70)**	-5.52 (-7.51 to -3.54)**	0.337	-2.48 (-3.21 to -1.75)**	0.342	-7.36 (-9.41 to -5.31)**	0.337
Arteriolar Tortuosity	1.71 (1.15-2.52)*	-1.02 (-3.10 to 1.06)	0.22	-0.45 (-1.23 to -0.33)	0.16	-1.12 (-3.35 to 1.12)	0.134
P-value†	**<0.001**	**0.002**		**<0.001**		**<0.001**	
cpFD	8.80 (3.93-19.70)**	-5.52 (-7.51 to -3.54)**	0.337	-2.48 (-3.21 to -1.75)**	0.342	-7.36 (-9.41 to -5.31)**	0.337
Venular Tortuosity	1.73 (1.15-2.59)*	-0.01 (-2.11 to 2.10)	0.215	-0.35 (-1.14 to 0.44)	0.157	-0.70 (-2.95 to 1.55)	0.13
P-value†	**<0.001**	**0.002**		**<0.001**		**<0.001**	
cpFD	8.80 (3.93-19.70)**	-5.52 (-7.51 to -3.54)**	0.337	-2.48 (-3.21 to -1.75)**	0.342	-7.36 (-9.41 to -5.31)**	0.337
Arteriolar Branching Angle	1.52 (1.01-2.30)*	-2.78 (-4.90 to -0.65)*	0.246	-1.10 (-1.90 to -0.30)*	0.19	-2.85 (-5.14 to -0.57)*	0.16
P-value†	**<0.001**	**0.016**		**0.004**		**0.001**	
cpFD	8.80 (3.93-19.70)**	-5.52 (-7.51 to -3.54)**	0.337	-2.48 (-3.21 to -1.75)**	0.342	-7.36 (-9.41 to -5.31)**	0.337
Venular Branching Angle	1.39 (0.96-2.01)	-2.69 (-4.72 to -0.66)*	0.247	-0.96 (-1.73 to -0.19)*	0.183	-2.67 (-4.86 to -0.48)*	0.158
P-value†	**<0.001**	**0.029**		**0.005**		**0.002**	

*denotes P<0.05; **denotes P<0.001; †Z-test for comparison of logistic regression models (leftmost column) and Steiger's Z-test for comparison of linear regression models (central and rightmost columns).

Retinal vascular metrics were analysed as per SD decrease. Results were adjusted for age, sex, axial length, systolic blood pressure, fellow caliber (for CRAE and CRVE), OCT signal strength (for linear regression analysis of average RNFL thickness) and OCT-A image score (for OCT-A capillary metrics).

ß = regression coefficient; CI = confidence interval; CRAE = central retinal artery equivalent; CRVE = central retinal vein equivalent; MD = mean deviation; OR=odds ratio; R^2 = adjusted coefficient of determination; RNFL = retinal nerve fiber layer; SAP = standard automated perimetry; VFI = visual field index.

Supplementary Table S6. Comparison of Current Findings in Relationships of Retinal Photographic Vascular Metrics and NTG with Previous Large Population-Based Eye Studies in POAG

**Retinal Photographic Vascular Metrics**	**BMES (high-pressure glaucoma: n = 38; low-pressure glaucoma: n = 21)**	**SiMES (POAG: n = 87)**	**Our Data (NTG: n = 100)**
Arteriolar Caliber	**Decreased (OR, 2.7; CI, 1.5-4.8)**	Not evaluated	No significant findings‡
Venular Caliber	Not evaluated	Not evaluated	No significant findings
Arteriolar D_f	Not evaluated	No significant findings	No significant findings
Venular D_f	Not evaluated	No significant findings	No significant findings
Arteriolar Tortuosity	Not evaluated	**Decreased (OR, 1.88; CI, 1.46-2.42)**	**Decreased (OR, 1.71; CI, 1.15-2.52)**
Venular Tortuosity	Not evaluated	**Decreased (OR, 1.61; CI, 1.28-2.02)**	**Decreased (OR, 1.73; CI, 1.15-2.59)**
Arteriolar Branching Angle	Not evaluated	No significant findings	**Decreased (OR, 1.52; CI, 1.01-2.30)**
Venular Branching Angle	Not evaluated	**Decreased (OR, 1.29; CI, 1.04-1.60)**	No significant findings

BMES = The Blue Mountains Eye Study;[11] CI = confidence interval; D_f = fractal dimension; NTG = normal pressure glaucoma; OR = odds ratio; POAG = primary open angle glaucoma; SiMES = The Singapore Malay Eye Study.[12]

‡In our study, we did not observe significant association between arteriolar narrowing and NTG after adjusting for age, sex, axial length, systolic blood pressure and fellow caliber. Previous studies provided evidences that in research relating to ocular outcomes, vessel calibers should be adjusted for axial length and modelled together to avoid confounding effects of increased axial elongation leading to stretching and thinning of retinal vasculature and the fellow component variable, respectively.[13,14] Contrary to our study, the Blue Mountains Eye Study did not adjust for axial length and fellow caliber in such analysis. We observed that using our data and only adjusting for age, sex and systolic blood pressure, we could also find significant associations between arteriolar narrowing and NTG (data not shown). Furthermore, such differences in results might also be attributable to the different computer programs used for analysis and the small sample size of low-pressure glaucoma patients (n = 21) in that study.

Supplementary Table S7. Intergrader Reliability of SIVA Image Analysis

	CRAE	CRVE	Arteriolar D_f	Venular D_f	Arteriolar Tortuosity	Venular Tortuosity	Arteriolar Branching Angle	Venular Branching Angle
ICC	0.897	0.928	0.706	0.729	0.678	0.628	0.798	0.802
95% CI lower bound	0.798	0.856	0.491	0.525	0.445	0.406	0.674	0.703
95% CI upper bound	0.954	0.968	0.859	0.871	0.861	0.863	0.932	0.945

CI = confidence interval; CRAE = central retinal arteriolar equivalent; CRVE = central retinal venular equivalent; D_f = fractal dimension; ICC = interclass correlation coefficient; SIVA = Singapore I Vessel Assessment.

Supplemental Table S8. Inter- and Intra-grader Reliability of OCT-A Image Analysis

Intergrader Reliability			Intragrader Reliability				
	cpVD	cpFD		cpVD		cpFD	
				Observer 1	Observer 2	Observer 1	Observer 2
ICC	0.996	0.973	ICC	0.993	0.994	0.984	0.990
95% CI lower bound	0.992	0.943	95% CI lower bound	0.985	0.988	0.967	0.979
95% CI upper bound	0.998	0.987	95% CI upper bound	0.997	0.997	0.993	0.995

CI = confidence interval; cpVD = circumpapillary vessel density; cpFD = circumpapillary fractal dimension; ICC = intraclass correlation coefficient.

References

1 Hubbard, L. D. *et al.* Methods for evaluation of retinal microvascular abnormalities associated with hypertension/sclerosis in the Atherosclerosis Risk in Communities Study. *Ophthalmology* **106**, 2269-2280, doi:10.1016/s0161-6420(99)90525-0 (1999).

2 Wong, T. Y. *et al.* Computer-assisted measurement of retinal vessel diameters in the Beaver Dam Eye Study: methodology, correlation between eyes, and effect of refractive errors. *Ophthalmology* **111**, 1183-1190, doi:10.1016/j.ophtha.2003.09.039 (2004).

3 Knudtson, M. D. *et al.* Revised formulas for summarizing retinal vessel diameters. *Curr Eye Res* **27**, 143-149 (2003).

4 Cheung, C. Y. *et al.* A new method to measure peripheral retinal vascular caliber over an extended area. *Microcirculation* **17**, 495-503, doi:10.1111/j.1549-8719.2010.00048.x (2010).

5 Mainster, M. A. The fractal properties of retinal vessels: embryological and clinical implications. *Eye (Lond)* **4 (Pt 1)**, 235-241, doi:10.1038/eye.1990.33 (1990).

6 Liew, G. *et al.* The retinal vasculature as a fractal: methodology, reliability, and relationship to blood pressure. *Ophthalmology* **115**, 1951-1956, doi:10.1016/j.ophtha.2008.05.029 (2008).

7 Stosic, T. & Stosic, B. D. Multifractal analysis of human retinal vessels. *IEEE Trans Med Imaging* **25**, 1101-1107 (2006).

8 Sasongko, M. B. *et al.* Alterations in retinal microvascular geometry in young type 1 diabetes. *Diabetes Care* **33**, 1331-1336, doi:10.2337/dc10-0055 (2010).

9 Cheung, C. Y. *et al.* Retinal vascular tortuosity, blood pressure, and cardiovascular risk factors. *Ophthalmology* **118**, 812-818, doi:10.1016/j.ophtha.2010.08.045 (2011).

10 Zamir, M., Medeiros, J. A. & Cunningham, T. K. Arterial bifurcations in the human retina. *J Gen Physiol* **74**, 537-548, doi:10.1085/jgp.74.4.537 (1979).

11 Mitchell, P. *et al.* Retinal vessel diameter and open-angle glaucoma: the Blue Mountains Eye Study. *Ophthalmology* **112**, 245-250, doi:10.1016/j.ophtha.2004.08.015 (2005).

12 Wu, R. *et al.* Retinal vascular geometry and glaucoma: the Singapore Malay Eye Study. *Ophthalmology* **120**, 77-83, doi:10.1016/j.ophtha.2012.07.063 (2013).

13 Liew, G. *et al.* Measurement of retinal vascular caliber: issues and alternatives to using the arteriole to venule ratio. *Invest Ophthalmol Vis Sci* **48**, 52-57, doi:10.1167/iovs.06-0672 (2007).

14 Li, L. J. *et al.* Relationship of ocular biometry and retinal vascular caliber in preschoolers. *Invest Ophthalmol Vis Sci* **52**, 9561-9566, doi:10.1167/iovs.11-7969 (2011).
